# Recurrent Fever of Unknown Origin (FUO) Due to Periodic Fever, Aphthous Stomatititis, Pharyngitis and Adenitis (FAPA) Syndrome in an Adult

**DOI:** 10.3390/jcm2030045

**Published:** 2013-08-19

**Authors:** Sigridh Muñoz-Gómez, Burke A. Cunha

**Affiliations:** 1 Infectious Disease Division, Winthrop-University Hospital, Mineola, New York, NY 11501, USA; E-Mail: sgomez@winthrop.org; 2 School of Medicine, State University of New York, Stony Brook, New York, NY 11794, USA

**Keywords:** adult FAPA syndrome, recurrent FUO

## Abstract

FAPA syndrome (periodic fever, aphthous stomatititis, pharyngitis and adenitis) is a relatively new entity described in pediatric patients. In adults, reports of FAPA are limited to rare case reports. The differential diagnosis of FAPA in adults includes Behcet’s syndrome, familial Mediterranean fever (FMF), Hyper IgD syndrome and juvenile rheumatoid arthritis (JRA), *i.e.*, adult Still’s disease. With FAPA syndrome, between episodes patients are completely asymptomatic and serologic inflammatory markers such as erythrocyte sedimentation rate (ESR), C-reactive protein (CRP) and white blood cell (WBC) count are normal. The etiology of FAFA is unknown, but lack of secondary cases or clustering in close contacts, lack of seasonality, and the lack of progression for years argue against an infectious etiology. We describe an extremely rare case of an adult with a recurrent FUO with profuse night sweats and prominent chills due to FAPA syndrome.

## 1. Introduction

Fever of unknown origin (FUO) was first defined by Petersdorf, but Knockaert later defined recurrent FUOs as recurrent FUOs with at least 2 weeks between febrile episodes. There are relatively few disorders, usually rheumatic/inflammatory, that cause periodic or recurrent FUOs, e.g., familial Mediterranean fever (FMF), Behcet’s syndrome. Rarely, other conditions, e.g., hypertriglyceridemia may cause recurrent FUOs [1,2] (Table 1).

**Table 1:** Differential diagnosis of recurrent fevers of unknown origin (FUOs). Adapted from [1,2].

**Rare Causes** Addison’s disease Adult Still’s disease (JRA) Behcet’s syndrome Chronic prostatitis Cirrhosis Cholesterol emboli Crohn’s disease (regional enteritis) Cyclic neutropenia Dental abscess (periapical) Drug fever Fabry’s disease Factitious fever Gaucher’s disease Granulomatous hepatitis Habitual hyperthermia Hemolytic anemia Hypertriglyceridemia Hypothalamic hypopituitarism Langerhan’s cell histiocytosis Lymphomas Malignant histiocytosis (histiocytosis X) Pulmonary emboli (small) Subacute bacterial endocarditis (SBE)—inadequately treated Subacute cholangitis Temporal arteritis (TA) Tuberculosis (TB) Whipple’s disease	**Very Rare Causes** Aorto-enteric fistula Atrial myxoma Castleman’s disease (multi centric) Chronic mastoiditis Chronic sinusitis Colon cancer Familial Mediterranean fever (FMF) Hyper-IgD syndrome Hypersensitivity pneumonitis (humidifier fever) Metal fume fever Polymer fume fever Pseudolymphoma Rosai-Dorfman syndrome Systemic mastocytosis
**Extremely Rare Causes** FAPA syndrome Erdheim-Chester disease Muckle-Well’s disease Schnitzler’s syndrome TNF-receptor-1-associated periodic syndrome (TRAPS)	

Recurrent FUOs are the most difficult of diagnostic challenges. Even after a detail focused diagnostic approach, many recurrent FUOs remain undiagnosed [1]. The value of repeated evaluations cannot be overestimated; findings not initially noticed may later become apparent, e.g., splenomegaly [2].

Marshall’s syndrome or FAPA (periodic fever, aphthous stomatititis, pharyngitis and adenitis) was firstdescribed in pediatrics [3]. In adults, reports of FAPA are rare [4,5]. The differential diagnosis of FAPA includes Behcet’s syndrome, Familial Mediterranean Fever (FMF), hyper-IgD syndrome [2,3]. Between episodes, FAPA patients are asymptomatic and laboratory tests are unremarkable. Lack of clustering, seasonality, and progression argue against an infectious etiology [4,5]. We report a case of recurrent FUO in an adult with profuse night sweats and prominent chills due to (FAPA).

## 2. Case

A 24-year-old male with recurrent FUOs was evaluated for recurrent fevers, chills and night sweats every two months for 3 years. When symptomatic, he has fevers to 103 F, sore throat, cervical lymphadenopathy and malaise for 3 days, followed by 3 days of profuse night sweats and prominent chills. Between episodes, heis completely asymptomatic. He is of Mediterranean descent. Repeated treatments, with antibiotics, colchicine, and non-steroidal anti-inflammatory drugs (NSAIDs) had no effect.

Examination was unremarkable, except for a single right posterior cervical node. His WBC count was 3.4 K/mm^3^, platelet count was 170 K/mm^3^, and ESR was 7 mm/h. Serum transaminases were normal, as was serum ferritin. HIV testing was negative. Coxsackie A/B titers, Toxoplasma, Bartonella, Epstein-Barr Virus (EBV) and cytomegalovirus (CMV) titers were negative. Serum protein electrophoresis (SPEP) was unremarkable. Anti-nuclear antibody and double stranded DNA titers were negative. Extensive work ups were non-diagnostic by oncology/hematology, infectious disease and rheumatology consultants but none inquired about aphthous ulcers. Since the patient had fever, cervical adenopathy and pharyngitis, suggesting FAPA, we asked him about aphthous ulcers during his febrile episodes. He reported aphthous ulcers occurred only during some attacks, but ulcers were not present with each attack.

## 3. Discussion

Recurrent FUOs remain a most difficult diagnostic challenge. The differential diagnosis in this case of recurrent FUO included cyclic neutropenia, hyper-IgD syndrome, Behcet’s syndrome, and FMF. Although of Mediterranean descent, he never had peritonitis, epididymitis, arthritis, and did not respond to colchicine suggesting FMF. Behcet’s was excluded with an unelevated ESR, no genital ulcers, and did not demonstrate pathergy with venipuncture. Causes of recurrent FUOs with cervical adenopathy, e.g., EBV, CMV, systemic lupus erythematosis (SLE), and toxoplasmosis were ruled out.

This case of recurrent FUO is particularly interesting because of the rarity in adults of FAPA. Because of his profuse night sweats, we were concerned about TB and lymphoma, but normal ESR, hemogram, SPEP and peripheral blood flow cytometry argued against lymphoma, and his T-spot was negative. FAPA syndrome can be difficult to diagnose because some clinical features may not present during attacks, e.g., aphthous ulcers as occurred with this case. His recurrent FUO was due to FAPA syndrome. He has been managed conservatively for a year, this is his fourth year of recurrent FUOs. FAPA syndrome responds to prednisone or cimetidine.

## 4. Conclusions

Clinicians should be aware that FAPA syndrome should be added to the list of rare causes of recurrent FUOs [1,2]. To the best of our knowledge this is the first reported case of recurrent FUO due to FAPA syndrome in an adult.
